# The mechanisms of humic substances self-assembly with biological molecules: The case study of the prion protein

**DOI:** 10.1371/journal.pone.0188308

**Published:** 2017-11-21

**Authors:** Gabriele Giachin, Ridvan Nepravishta, Walter Mandaliti, Sonia Melino, Alja Margon, Denis Scaini, Pierluigi Mazzei, Alessandro Piccolo, Giuseppe Legname, Maurizio Paci, Liviana Leita

**Affiliations:** 1 Department of Neurosciences, Scuola Internazionale Superiore di Studi Avanzati (SISSA), Trieste, Italy; 2 Department of Chemical Sciences and Technologies, University of Rome “Tor Vergata”, Rome, Italy; 3 School of Pharmacy, East Anglia University, Norwich, United Kingdom; 4 CREA Consiglio per la ricerca in agricoltura e l’analisi dell’economia agraria (Council for Agricultural Research and Economics), Gorizia, Italy; 5 Life Science Department, University of Trieste, Trieste, Italy; 6 ELETTRA Synchrotron Light Source, Trieste, Italy; 7 Interdepartmental Research Centre (CERMANU), University of Naples Federico II, Napoli, Italy; University of Verona, ITALY

## Abstract

Humic substances (HS) are the largest constituent of soil organic matter and are considered as a key component of the terrestrial ecosystem. HS may facilitate the transport of organic and inorganic molecules, as well as the sorption interactions with environmentally relevant proteins such as prions. Prions enter the environment through shedding from live hosts, facilitating a sustained incidence of animal prion diseases such as Chronic Wasting Disease and scrapie in cervid and ovine populations, respectively. Changes in prion structure upon environmental exposure may be significant as they can affect prion infectivity and disease pathology. Despite its relevance, the mechanisms of prion interaction with HS are still not completely understood. The goal of this work is to advance a structural-level picture of the encapsulation of recombinant, non-infectious, prion protein (PrP) into different natural HS. We observed that PrP precipitation upon addition of HS is mainly driven by a mechanism of “salting-out” whereby PrP molecules are rapidly removed from the solution and aggregate in insoluble adducts with humic molecules. Importantly, this process does not alter the protein folding since insoluble PrP retains its α-helical content when in complex with HS. The observed ability of HS to promote PrP insolubilization without altering its secondary structure may have potential relevance in the context of “prion ecology”. These results suggest that soil organic matter interacts with prions possibly without altering the protein structures. This may facilitate prions preservation from biotic and abiotic degradation leading to their accumulation in the environment.

## Introduction

Humic substances (HS) are the product of animal, plant, and bacterial tissue decay and comprise the major fraction of natural organic matter ranging from 60–70% of total organic carbon in soils [1]. HS are supramolecular associations of small heterogeneous molecules self-assembled by weak forces and hydrogen bonds [2]. The conformational arrangement of HS is known to control their interaction with other components in the environment, but these processes are not well understood [3]. HS have been known for some time to facilitate the environmental transport of hydrophobic organic molecules, metal contaminants and radionuclides [4]. Also complexation of HS with oppositely charged polyelectrolytes and surfactans has been investigated [5,6]. Natural proteins in solution are readily biodegradable, but they may be preserved by interacting with humic molecules [7]. Adsorption to HS may affect the fate of toxic and infectious proteins, including for instance insecticidal proteins or prions [8,9]. Protein adsorption to humic molecules may potentially lead to their accumulation in the environment.

Despite its relevance, the mechanisms of protein interaction with HS are still not completely understood. The goal of this work is to advance a structural-level picture of the encapsulation of an intact protein by natural HS. As a model system we used recombinant α-helical folded prion protein (PrP) adsorbed to different HS -humic (HA) and fulvic (FA) acids- extracted by different terrestrial sources.

The PrP (or PrP^C^ in its physiological and cellular form) is a highly conserved protein mostly expressed in the central and peripheral nervous systems. The mature PrP^C^ is composed of 208 residues including a largely unstructured N-terminal part and a globular α-helical C-terminal domain [10]. Its biological activity is still far from being clear but there is enough evidence that PrP^C^ plays a role in several physiological functions in the nervous systems [11]. In the brain PrP^C^ may adopt an amyloidogenic, partially protease-resistant conformation enriched in β-sheet secondary structures known as prion or PrP^Sc^ [12], which is related to a class of human and animal neurodegenerative diseases denoted as transmissible spongiform encephalopathies (TSE) including scrapie in sheep and goat, chronic wasting disease (CWD) in cervids and bovine spongiform encephalopathy (BSE) in cattle [13].

Scrapie and CWD are of particular environmental concern as they are horizontally transmissible and remain infectious after years in the environment [14,15]. In ruminants, PrP^Sc^ can be excreted or secreted within different biological materials -mainly feces, urines, saliva, skin and placenta- and released into soil and water [16]. A recent report showed that plants can potentially adsorb and transport infectious prions [17]. Cervids and other animals likely consume prions contained in these reservoirs and become infected. Clay-bound prions may retain infectivity, as experimentally validated in several *in vivo* prion infection studies [18–24]. This evidence supports a role for the soil as stable reservoir of infectious PrP^Sc^ posing a considerable environmental concern.

In addition to the mineral components, the soil environment contains native organic matter, whose major constituents are HS. Thus, it is important to consider also the role that HS play in protein adsorption. Studies on interaction between recombinant PrP and HS have shown that HS promote PrP adsorption forming insoluble complexes [25–28]. Previously, we showed that HS are potent protein complexing agents that can form insoluble, protease resistant assemblies with PrP. Interestingly, HS may act as anti-prion agents in prion infected neuronal cells and in the amyloid seeding assays [29]. At that time, we interpreted our findings as environmentally relevant, as the interactions of prions with HS may potentially affect their availability. Although not confirmed in natural environment, this hypothesis found supports in previous study in animal models showing that prions bound to a soil rich in HS are less infectious than PrP^Sc^ adsorbed to montmorillonite [19]. Another study has reported that HA-adsorbed PrP^Sc^ strains resulted slightly less infectious when intracerebrally inoculated in hamsters [30]. However, if HS-bound prions retain infectivity upon oral ingestion in ruminants and in cervids is still debated [20].

The structural mechanisms of protein-HS interaction remain still controversial. As HS are composed by a complex self-assembling arrangement of relatively small molecules with a prevalent negatively charged character and mainly carboxylic (fatty acids) and phenolic-types (lignin residues) as acidic functional groups [31], protein interactions may be reasonably mediated by both charged and hydrophobic moieties. Here, we used structural biology approaches, such as solution-state NMR and Fourier transform infrared spectroscopy (FTIR), to describe the self-assembly of different HS in the presence of a macromolecule as recombinant, non infectious, PrP. We observed that PrP precipitation upon adsorption to HS is mainly driven by a mechanism of “salting out” whereby PrP molecules are rapidly removed from the solution and aggregate in insoluble adducts with HS. This process does not alter the protein folding since insoluble PrP retains its α-helical content when in complex with HS as shown by FTIR. A divalent cation, zinc, has been also investigated in our NMR experiments showing a synergistic effect with HS in inducing PrP precipitation.

The observed ability of HS to promote PrP adsorption without altering its secondary structure may have potential relevance in the context of “prion ecology”. Soil organic matter may adsorb proteins, as PrP^Sc^, promoting prions preservation from biotic and abiotic degradation and leading to their accumulation in the environment.

## Materials and methods

### Characterization of humic substances

The HS used in this study consisted in three humic acids (HA) and three fulvic acids (FA) extracted and isolated from a green compost made only of cauliflower wastes (namely, HAGw and FAGw, respectively), sandy loam soil (HAS and FAS, respectively) sampled in Gorizia (N-E Italy), HA from Leonardite (HLe) and fulvic acids from tomato biowastes (FABw). The humic materials were extracted, separated and purified as reported elsewhere [32]. After purification the extracts were dialyzed against deionised water until Cl-free and freeze-dried. The specifications and abbreviations used are reported in Table 1. The elemental composition (C, H, N) was determined by an elementary analyzer (Fisons Interscience EA118) with 2 mg of each HS. The carbon distribution in HS as percent of carbon content was obtained by solid-state Cross Polarization Magic Angle Spinning (CPMAS) NMR spectroscopy on a Bruker Avance operating at 300 MHz on ^13^C at 75.4 MHz. The HS were packed in zirconia rotors with KelF caps with rotation speed of 12–13,000 Hz using a contact time of 1.0 ms. Four thousand scans were accumulated with 2.0 s of relaxation delay.

**Table 1:** Samples of humic substances used in this study.

Name and source	Abbreviation
Humic acid extracted from Greenwaste^1^	HAGw
Fulvic acid extracted from Greenwaste^1^	FAGw
Soil humic acid^2^	HAS
Soil fulvic acid^2^	FAS
HA Leonardite^3^	HALe
Fulvic acid extracted from Bio waste^4^	FABw

^1^ HA, FA extracted from green compost made only of cauliflower wastes.

^2^ HA, FA extracted from sandy loam Mollic Hapludalf (USDA) soil.

^3^ HA extracted from North Dakota Leonardite.

^4^ FA extracted from compost made only of tomato wastes

### Recombinant PrP expression and purification

The murine full-length PrP -MoPrP(23–230)- and the N-terminally truncated form -MoPrP(89–230)- were expressed, purified and *in vitro* refolded in buffer 25 mM sodium acetate at pH 5.5 according to our previous protocols [33,34]. For isotope labelling 1 g/L [^15^N] ammonium chloride were added to minimal medium where bacteria grew as previously described [35].

### NMR spectroscopy interaction studies on MoPrP with HS

All ^1^H NMR spectra were performed with Bruker Avance Instruments operating at 700 MHz using the zgpr pulse program of the Bruker library for water signal suppression with a relaxation delay of 2 s. ^1^H NMR spectra of MoPrP(89–230) and of MoPrP(23–230) at 90 μM were acquired separately with 256, 512 and 2500 scans. A series of ^1^H NMR spectra on MoPrP(89–230) and MoPrP(23–230) at 90 μM were performed in presence of increasing amounts of HAGw (60, 120 and 180 μg/mL) and FABw (60, 120, 180 and 240 μg/mL) with 2500 scans. ^1^H NMR spectra of MoPrP(23–230) at 300 μM alone and in presence of increasing amount of ZnCl_2_ (100, 400, 1000 μM) were collected with 16 scans. NMR spectra of MoPrP(89–230) and of MoPrP(23–230) uniformly ^15^N-labeled alone and in the presence of HA, FA and ZnCl_2_ were run at 298 K on a Bruker Avance instrument operating at 700 MHz. ^1^H−^15^N HSQC NMR spectra of MoPrP(89–230) at 160 μM in absence and presence of FABw (120 μg/mL) were acquired with 16 scans. The two-dimensional NMR experiments were performed in phase sensitive mode with a time proportional phase increment (States-TPPI) [36,37] typically using 4K of memory for 380 increments.

Diffusion NMR spectra for MoPrP(23–230) at 300 μM alone and in presence of increasing amount of ZnCl_2_ (100, 300 and 600 μM) were acquired. The DOSY experiments [38] were performed using the ledbpgppr2s pulse sequence with presaturation for water signal suppression and collecting 32 monodimensional spectra with 64 scans in a linear increasing gradient from 5% to 95% with a Δ of 70 ms and a δ of 2 ms. The TOPSPIN 3.1 software package was used for data processing and analysis.

### Infrared spectroscopy of the MoPrP-HS precipitates

The precipitated samples of MoPrP(23–230) (1.2 mg) treated with the HS (120 μg/mL) were isolated by centrifugation while the supernatant was discarded. Samples were then lyophilized overnight and used for FTIR spectrum acquisition on a Perkin Elmer FTIR spectrometer on NaCl_2_ crystal discs. All data were collected using 128 scans at a resolution of 4 cm^−1^. HS FTIR spectra in the absence of MoPrP(23–230) collected in the same conditions were subtracted from the raw spectra acquired for each protein treated sample. Then the set of the data obtained were analyzed in the region 1710–1590 cm^−1^ (amide I band) by normalizing the spectra and the second derivate for each sample was carried out in order to compare qualitatively and quantitatively the secondary structure of the precipitated MoPrP(23–230) as previously described [39,40,41].

### Atomic force microscopy of MoPrP-HS complexes

MoPrP(23–230) at 25 μg/mL were incubated with 5 μg/mL of different HS in 50 μL volume at room temperature for 6 hours. All the samples were prepared by drop casting on a surface of freshly cleaved muscovite mica and left to adhere till solvent evaporation. All AFM measurements were performed as previously described [29].

## Results

### Chemical features of humic and fulvic acids

The elemental composition of HS samples is shown in S1 Table. As expected, the humic acids (HAGw, HAS and HALe) resulted richer in carbon and nitrogen than fulvic acids while the hydrogen content was similar. The HALe sample from North Dakota lignite appeared to be the richest in both carbon and nitrogen. The CPMAS-^13^C-NMR spectra allowed to obtain the carbon distribution present in each sample as percent of carbon content (S2 Table). The humic acids showed the greatest content of alkyl and aromatic carbon, while the fulvic acids were generally richer in carboxyl group [42]. This distribution of carbon compounds determined that HALe was the most hydrophobic humic material followed, in the order, by HAGw > FAS > HAS ≥ FAGw > FABw (S2 Table).

The NMR spectra of humic or fulvic acids in solution confirmed the results obtained by solid-state NMR spectroscopy. The HALe sample showed the largest content of aromatic groups and the smallest of hydroxyalkylic moieties. No significant differences were observed in HAGw and HAS samples, except for data recorded in the region around 3–5 ppm where the spectrum of hydroalkylic protons of HAGw is comparatively more populated than that of HAS. The liquid-state NMR spectra in solution of fulvic acids (data not shown) revealed a larger content of resonances in the region between 3–5 ppm due to saccharidic compounds of vegetal origin as confirmed by the presence of the intense resonances at 100 ppm due to anomeric carbons in the ^13^C NMR spectra. Moreover, aromatic resonances and the intense resonance at 2.3 ppm evidenced the methylenic groups attributable to fatty acids and lignins [2,31,43]. The solid-state high resolution NMR spectroscopy experiments performed on HS samples showed different concentrations in negatively charged carboxilic functional groups. These carbon resonances intensities are approximately proportional to the number of functional groups present in the HS employed here (S2 Table). We found that the larger number of carboxylic (polar) groups are present in FABw (7.92%), FAS (9.50%) and HAS (10.57%) samples. These resonances are due to carboxyl groups on relatively hydrophobic molecules, which are likely the most effective in the interaction with proteins and able to alter the equilibria between solvent-solvent and solvent-solute interaction leading to protein precipitation.

The solution-state NMR spectra showed different molecular flexibility of HS. The fine structure superimposed in the spectra of the HS indicated that HAGw and HAS feature a flexible structure while HALe are more compacted and stiffened. Among fulvic substances, FABw and FAS appear more flexible than FABw. Interestingly, FAS showed flexibility also in the aromatic region differently from the other samples. Results of the molecular diffusion of HS by DOSY indicated different profiles in a range of diffusivity from logD = -10.1 to -9.85 (m^2^/s) for the samples HAGw, HAS and HALe. As HS are supramolecular assemblies stabilized by weak interactions, the study on diffusion of these substances is a powerful instrument to monitor the aggregation levels of HS in the presence of ligands [44]. The results obtained from our investigations also showed that HAS displayed the greatest diffusivity compared to HAGw and HALe. It is possible that the larger content of aromatic moieties in HAS may favour a hydrophobic group-mediated aggregation process when in the presence of ligands such as proteins. The DOSY NMR diffusion spectra of fulvic acids revealed different diffusion profiles in a range of diffusivity (log D) from -9.9 to -9.65 m^2^/s, with the following order of diffusivity: FAS > FAGw > FABw. In these samples, the differences in diffusivity between aromatic and hydroalkyl moieties may suggest a tendency of aggregation due to hydrophobic interactions, particularly in FAGw and FABw.

### AFM characterization of PrP-HS assemblies

**Fig 1:**
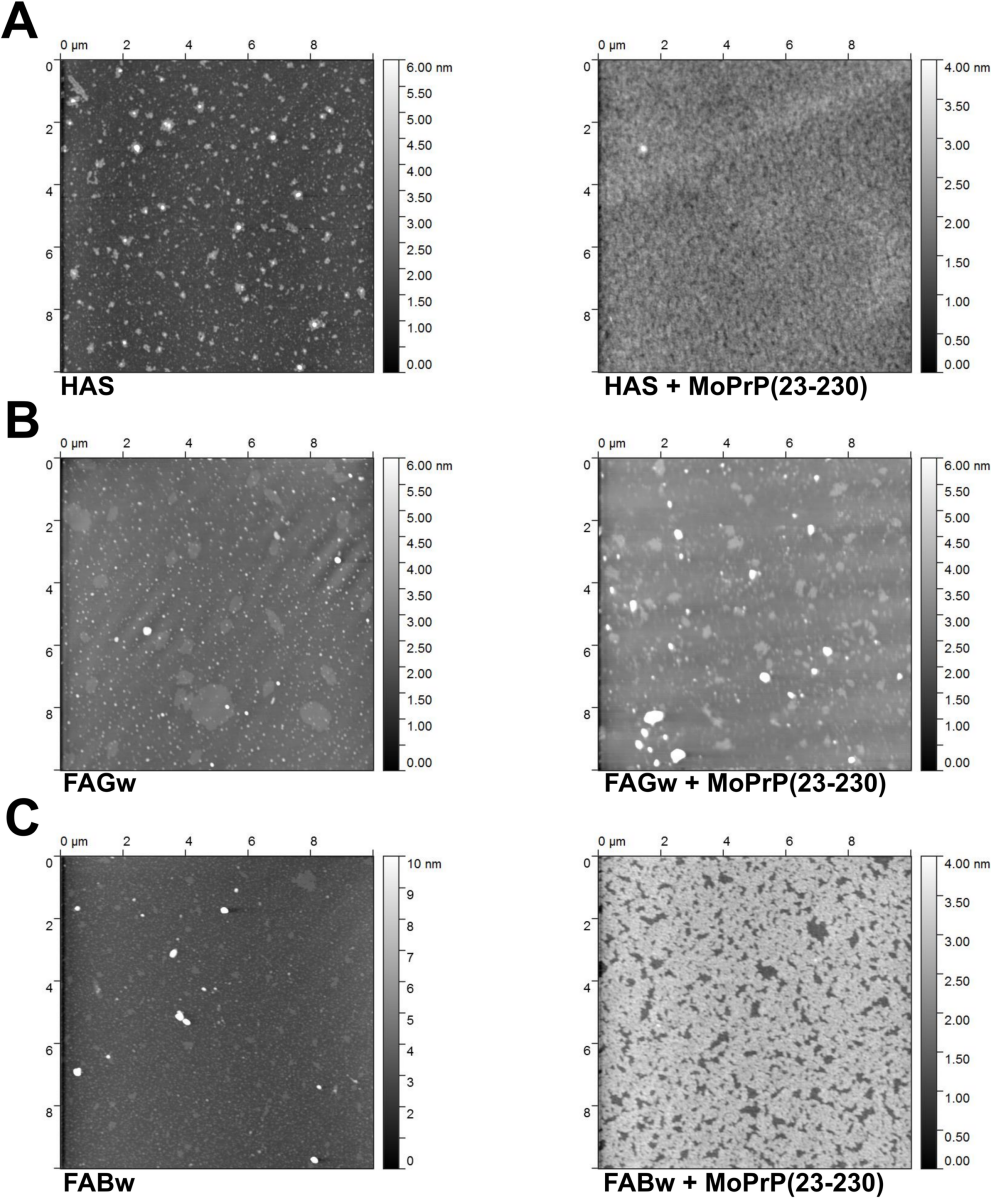
AFM characterization of HS and MoPrP-HS complexes. Surface morphology of HS alone (HAS, FAGw and FABw in panels A, B and C left side) and HS complexes with 25 μg/mL of MoPrP(23–230) (right side); MoPrP(23–230) alone is shown in S1 Fig, panel A.

To characterize the PrP-HS assemblies we analyzed their morphology by AFM. This technique has been employed to study the topography and conformational structures of HS on solid surface as well as protein complexes formed with soil or HS [45,46,47]. The AFM scans showed initial rearrangements of the assemblies formed upon addition of HS. The HS here investigated revealed a heterogeneous morphology, with large aggregate assemblies possibly attributed to their different physico-chemical nature. As previously observed [29], the adsorption of MoPrP(23–230) to HS seems to affect the overall distribution of the adducts on the mica surface. After MoPrP adsorption into HS, the assemblies became more compact and heterogeneous, forming supramolecular clusters with height and length between 4 nm and 100 nm (S1 Fig and Fig 1). Interestingly, in the presence of MoPrP, FABw associates in a narrowed and small ordered assemblies (Fig 1C) confirming the ability of the FA-MoPrP complex to arrange in regular structures.

### Solution-state NMR spectroscopy on PrP titrated with HS

**Fig 2:**
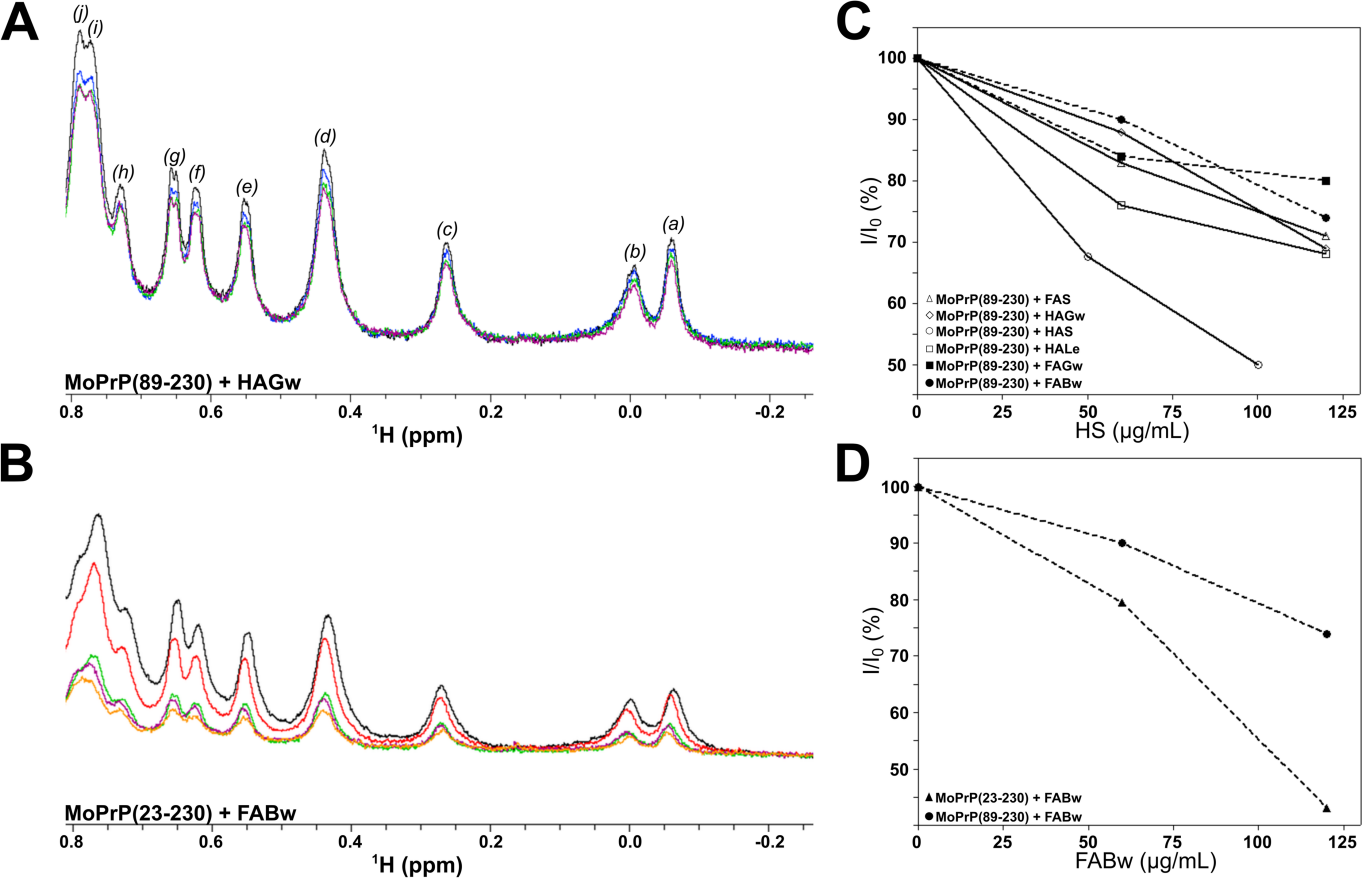
Titration of MoPrP with HS monitored by NMR. In panel A, region of 1D-NMR spectrum of ring current shifted methyl resonance (RCSMs) of MoPrP(89–230) at 90 μM in 5% D_2_O at increasing concentration of HAGw. Colored in black: MoPrP(89–230) alone; blue: in presence of 60 μg/mL HAGw; green: in presence of 120 μg/mL HAGw; violet: in presence of 180 μg/mL HAGw. The assignments were assigned according to BMRB NMR data bank (id 17174) as follows: I139 (*a*); L130 (*b*); L182 (*c*); I139 (*d*); V161 (*e*); L125 and L130 (*f*); R156 (*g*); L125 and V166 (j); I184 and T201 (*i*). In panel B, region of the NMR spectrum (RCSM) resonances of MoPrP(23–230) at 90 μM in 5% D_2_O. In black: MoPrP(23–230) alone; red: in presence of 60 μg/mL FABw; green: in presence of 120 μg/mL FABw; violet: in presence of 180 μg/mL FABw; orange: in presence of 240 μg/mL FABw. In panel C, precipitation of MoPrP(89–230) in presence of HS: FABw (dashed line with black circle), FAGw (dashed line with black square), HALe (continuous line with open square), HAS (continuous line with open circle), HAGw (continuous line with open diamond) and FAS (continuous line with open triangles). In panel D, precipitation of MoPrP(89–230) (dashed line with black circle) and MoPrP(23–230) (dashed line with black triangles) in presence of FABw.

MoPrP(89–230) and MoPrP(23–230) were titrated with HS purified from different organic matrices. We noticed that the addition of increasing concentration of HS caused precipitation of both the truncated and full-length MoPrP. The results obtained from spectroscopic investigations revealed a common pattern of MoPrP-HS interaction. As significant example, we show the ^1^H NMR spectra of the dynamics of interaction between MoPrP (truncated or full-length) and HAGw or FABw (Fig 2). The NMR spectra were analyzed in the “ring current shifted methyl” protons region (RCSMs, *i*.*e*. -1 to -2 ppm) where the resonances of the protons of methyl side chain of amino acid located in the folded region of the protein, close to magnetic deshielding groups at the edge of aromatic rings, are observed. It is agreed that the shielding effect of these resonances is strictly associated to the structural feature of the proteins and therefore is considered an extremely sensitive indicator for assessing the stability of the tertiary structure. Observation of large effects on resonances intensity and chemical shift in this spectral region (RCSMs-NMR region) are also diagnostic of structural changes including also partial unfolding. The resonances of the RCSMs-NMR spectra of both the MoPrP(89–230) and MoPrP(23–230) revealed a similar pattern after the additions of HAGw or FABw (Fig 2A and 2B, respectively). An accurate inspection of ^1^H NMR data showed that the resonances shown in Fig 2, panel A, belong to the methyl groups present in the globular and N-terminal domains of MoPrP(89–230) as reported in the BMRB NMR data bank (id 17174) [48].

The decrease of the intensity of the resonance without any change of chemical shift of these resonances after the HS addition suggests that the interaction leads to the formation of insoluble adducts without altering the native folding of the protein in solution neither the conformation of the flexible and unfolded domain (residues 23–127). In fact, the absence of any distortion of the spectrum as resonance broadening and/or change of chemical shift led to exclude the interaction between soluble forms and the occurrence of fast or intermediate exchange between the bound and free forms of the proteins in solution indicating that the precipitation was the exclusive process involved.

Beside the common mechanism of protein-humic substances interaction, the quantitative efficiency of the protein precipitation was strictly dependent on the intrinsic characteristics of HS used here. Our results show that fulvic acids (FABw and FAGw) were more effective in inducing protein precipitation (Fig 2C and 2D).

**Fig 3:**
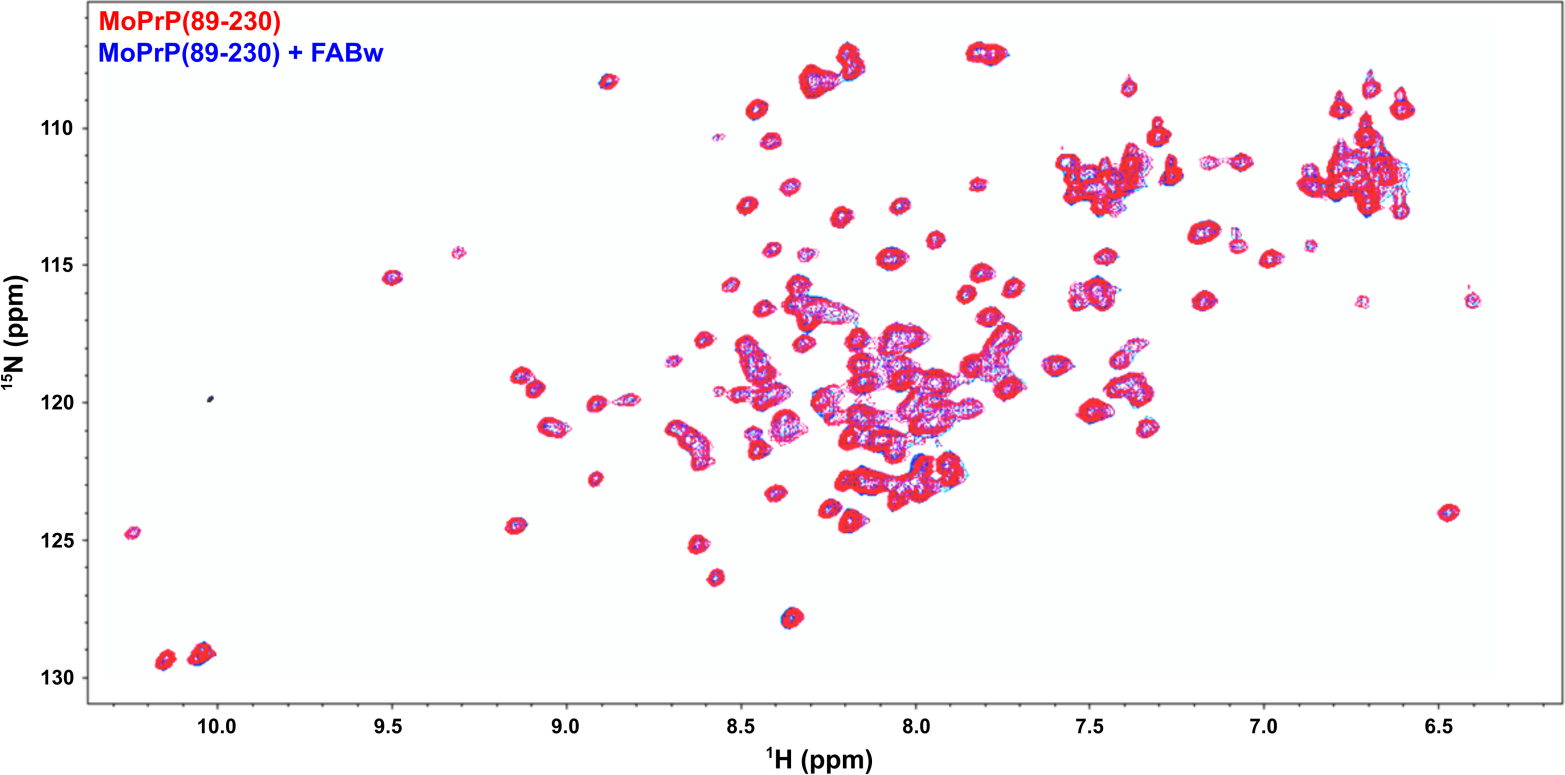
^1^H-^15^N HSQC spectrum of MoPrP(89–230) in the presence of a fulvic acid. Superimposition of the the spectra of the protein alone (in red) and after the addition of FABw (in blue).

To confirm these results and to monitor changes in the MoPrP secondary structure, the interaction between uniformly labelled ^15^N-MoPrP(89–230) and a fulvic acid, FABw, has been performed. The ^1^H-^15^N HSQC spectrum shows good dispersion of amide signals indicating that the protein has the same native conformation as previously observed [49,50]. Upon addition of FABw to MoPrP(89–230) no chemical shift perturbation was detectable (Fig 3). A decrease of intensity of the cross-peaks due to protein precipitation was observed.

**Fig 4:**
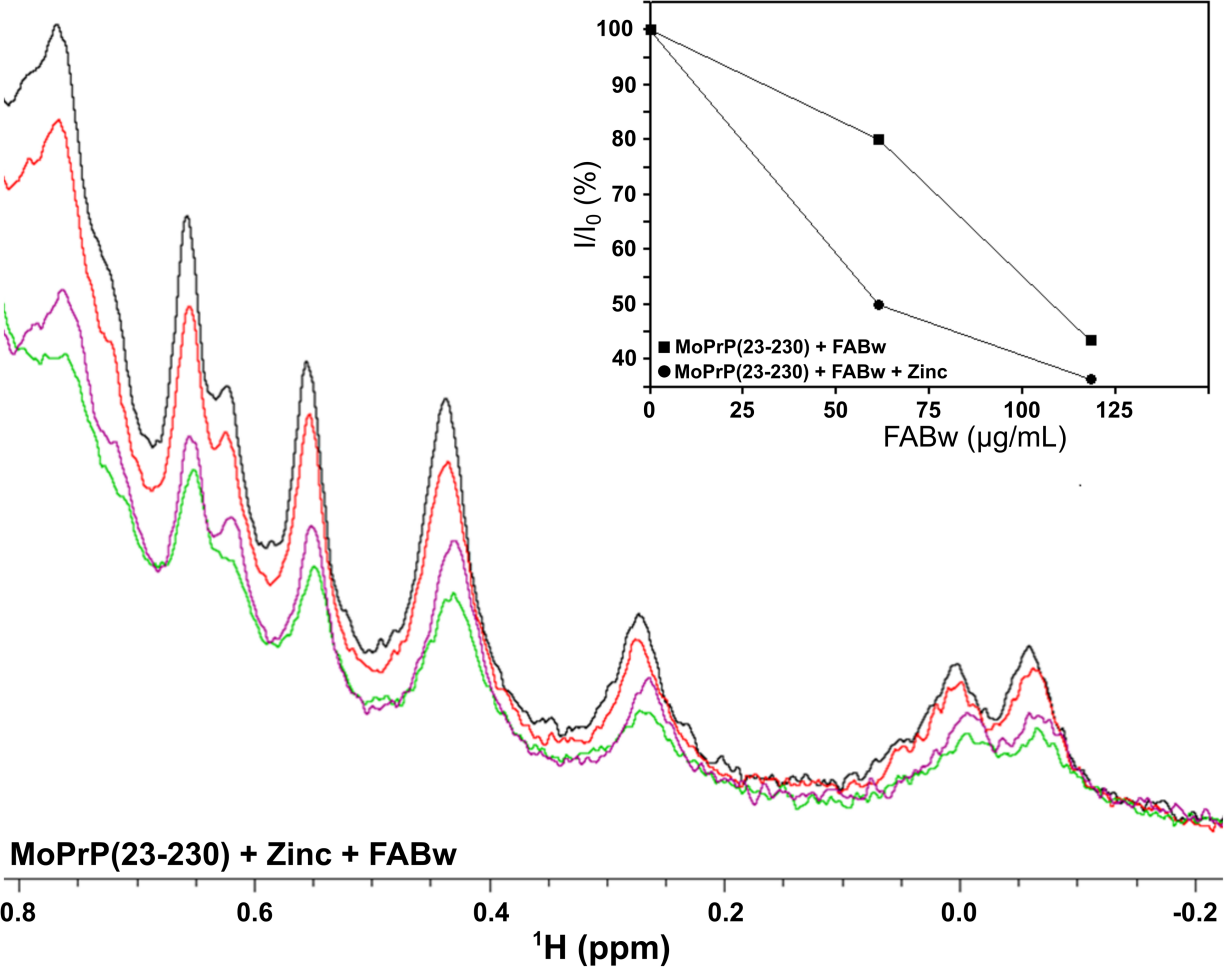
Titration of MoPrP(23–230) with a fulvic acid and Zn^2+^ monitored by NMR. Combined effect upon the addition to MoPrP(23–230) of Zn^2+^ and FABw. Enlarged region of high field shifted methyl resonances of the 1D-NMR spectra of unlabeled MoPrP(23–230) upon addition of Zn^2+^ and FABw. In black: MoPrP(23–230) alone; in red, MoPrP(23–230) with ZnCl_2_ (always at 90 μM); in green, MoPrP(23–230) with ZnCl_2_ and 60 μg/mL FABw; in violet, MoPrP(23–230) with ZnCl_2_ and 120 μg/mL FABw; in yellow, MoPrP(23–230) with ZnCl_2_ and 180 μg/mL FABw. In the *inset*, differences in resonance intensities of MoPrP(23–230) due to the precipitation in the presence of the FABw alone (filled square) or in the presence of both FABw and Zn^2+^ (filled circle).

PrP^C^ can bind copper and zinc cations through the metal binding sites present in the unstructured N-terminal domain, *i*.*e*. four octarepeats (of sequence PHGGGWGQ) and the so called fifth copper binding site (residues 90–111). Specifically, one Zn^2+^ is coordinated by four octarepeats [51,52,53]. The diamagnetic property of Zn^2+^ allows to obtaining clear NMR data without broadening effects that may occur in the presence of paramagnetic ions such as Cu^2+^. The addition of Zn^2+^ to MoPrP(23–230) is shown in the S2 Fig where the region of the spectrum of RCSMs resonances is displayed. The decrease of the intensity of the resonances indicates a clear precipitation process occurring without any significant conformational change upon binding to Zn^2+^. To exclude the possibility that the full-length MoPrP may form multimeric species in the presence of Zn^2+^ we performed Diffusion Ordered Spectroscopy (DOSY) which allows to separate the spectra of chemical species which have different hydrodynamic radii (S3 Fig). The diffusion fronts of the DOSY experiments showed no clear changes, thus indicating that Zn^2+^ ions do not promote protein aggregation and polymerization. In the presence of both FABw and Zn^2+^ we observed an increased insolubility of MoPrP(23–230) suggesting an additive effect of HS and the metal in promoting protein precipitation (Fig 4).

### Infrared spectroscopy studies on MoPrP-HS insoluble adducts

**Fig 5:**
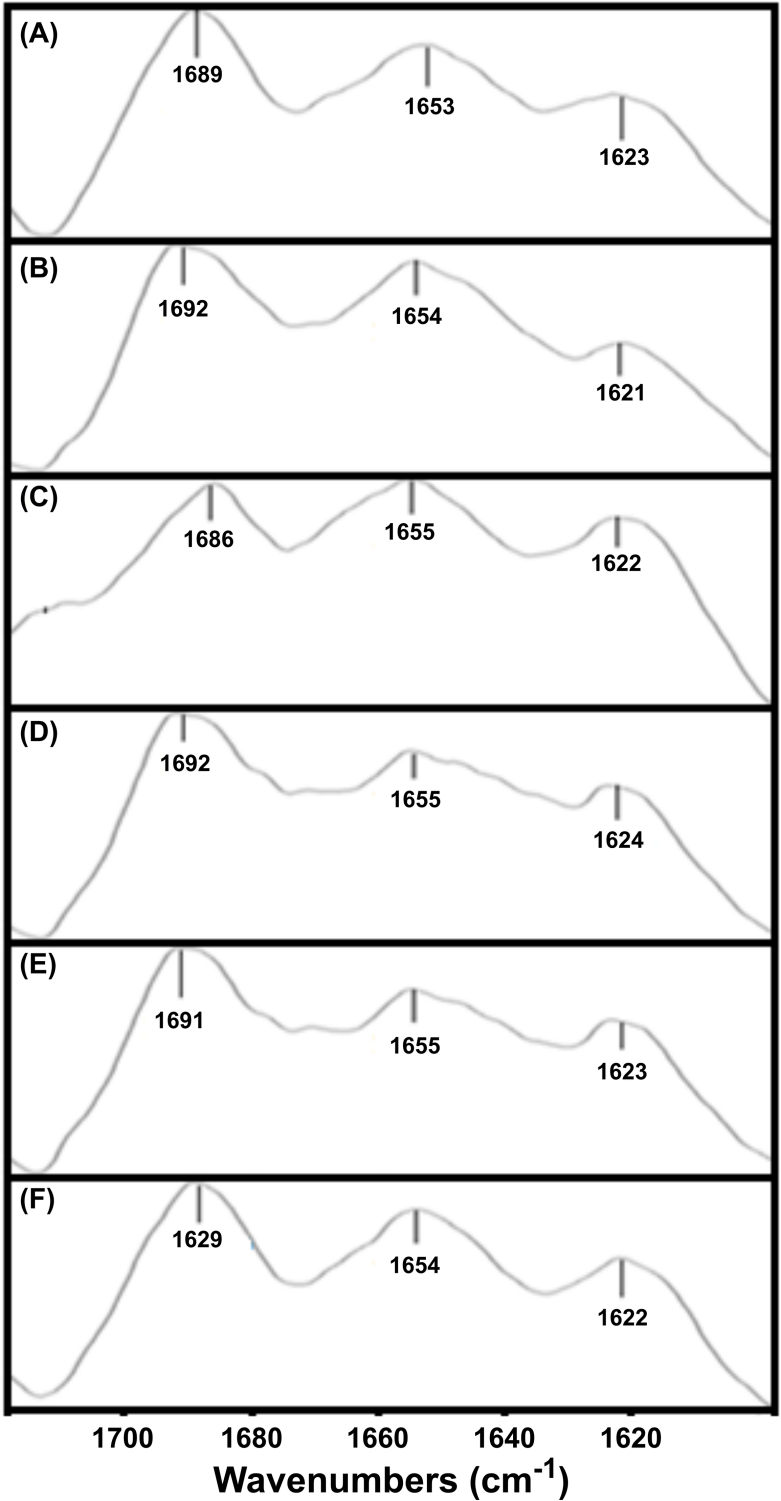
FTIR spectra of the MoPrP(23–230) precipitates in complex with different HS. The tested HS were at 120 μg/mL, all the results are reported in Table 2.

The second derivative of the FTIR spectrum is indicative of the ratio between the secondary structure elements in the precipitated protein samples. We analyzed the amide I region of the FTIR spectrum (1700–1600 cm^-1^), which corresponds to the absorption of the carbonyl peptide bond group of the protein main chain. Deconvolution of FTIR spectra allowed us to assign the individual secondary structure elements of incubated MoPrP(23–230) with HS and their relative contribution to the main absorbance signal (Fig 5 and Table 2). In all cases, bands at ∼1689 cm^-1^, ~1653 cm^-1^ and ~1623 cm^-1^ -corresponding to turns, α-helices and β-sheet secondary structures, respectively [54,55]- dominate the spectra without any significant differences between MoPrP(23–230) alone and in complex with HS. The results from IR experiments indicate that MoPrP(23–230) structure in complex with HS did not changes in the solid state precipitates, thus confirming that HS barely affect the protein structure upon interaction.

**Table 2:** Results of the fitting of the second derivative of the IR spectra of the MoPrP(23–230) in complex with HS.

Samples	Peak at (cm^-1^)	Secondary structures (SS)	% of SS
**MoPrP (A)**^1^	1689	Turn	36
	1653	α-helix	33
	1623	β-sheet	31
**MoPrP + FABw (B)**	1692	Turn	40
	1654	α-helix	36
	1621	β-sheet	34
**MoPrP + FAGw (C)**	1686	Turn	39
	1655	α-helix	41
	1622	β-sheet	30
**MoPrP + HAGw (D)**	1692	Turn	28
	1655	α-helix	39
	1624	β-sheet	33
**MoPrP + HAS (E)**	1691	Turn	39
	1655	α-helix	33
	1623	β-sheet	28
**MoPrP + HALe (F)**	1689	Turn	39
	1654	α-helix	34
	1622	β-sheet	27

^1^ The letters refer to the FTIR spectra represented in Fig 5

## Discussion

In this work we provided a structural characterization of the interaction between the non-infectious PrP and humic substances extracted from different organic matrices. We monitored by solution-state NMR spectroscopy the progressive precipitation of the PrP in the presence of HS and we proved that such interaction occurs without any substantial conformational changes to the protein. Moreover, the combined effect of the addition of zinc and fulvic acid to MoPrP(23–230) gave clearly indications that the metal cooperates in the precipitation mechanism without affecting the protein conformation. This may indicate that the mixed polyelectrolytic/hydrophobic nature of the molecular components of HS can interact with complementary sites of the MoPrP, thus removing the water on the protein surface and causing its precipitation. The common feature between HA and FA is that both HS cause precipitation in the same way but with different extent, presumably depending on the intrinsic nature of the HS, as already postulated [29]. These observations lead to the conclusion that the process is local, non specific and involves the polar and the hydrophobic regions of the protein. This mechanism may be interpreted as “salting out” of the protein by the electrostatic perturbation induced by the polar moieties of HS. This well known phenomenon is based on the electrolyte/non-electrolyte interactions where the non-electrolyte is less soluble at high salt concentration [56]. The NMR spectra on MoPrP showed that the resonances decrease their intensities with stable, unperturbed chemical shifts and line-widths during the HS addition.

It is possible to postulate a general mechanism whereby the addition of HS produces a modification of some sites of the protein due to both the polar and hydrophobic functions of the humic molecules, which are able to alter the solvation shell of protein leading to precipitation. This effect is very general, with different extent due to the molecular differences present in the HS. In particular, fulvic acids -as FAGw and FABw- are more effective in inducing MoPrP precipitation suggesting that their polarity, in terms of carboxyl groups number and flexibility, may play a complex role in the capacity of interaction with proteins.

FTIR spectroscopy indicates that any conformational changes occur in the secondary structure of the protein upon precipitation with the HS. This suggests that a simple process of “salting out” occurs without any specific intermolecular recognition and alteration of the secondary structures of the MoPrP protein, as already reported for other proteins [57,58]. This clearly indicated that the precipitation of MoPrP by HS can be considered as a common mechanism observed with different proteins in the absence of a specific interaction and specific recognition beyond the polar interaction(s) between proteins and polar substances and water [59]. The electrolytic/hydrophobic nature of humic molecules exerts behaviour of structured ions which are able to produce this particular form of “salting out” without changing the secondary structure of the protein. The several carboxylic groups present in the HS are likely to play an active role in the interaction with protein sites.

Here, we used natively folded full-length and truncated MoPrP to test the interaction with natural HS. Clearly, a more environmentally relevant model would be structural studies on PrP^Sc^-HS complexes, warranting further investigations. Changes in PrP^Sc^ structure upon environmental exposure may be as significant as changes in PrP^Sc^ quantity and bioavailability, since the PrP^Sc^ structure can directly affect infectivity and disease pathology [60]. The observation that HS do not affect the protein folding suggests that, despite the interactions with humic molecules, the correct folding of infectious prions is likely preserved also from biotic and abiotic degradation in natural conditions, thus leading to their accumulation in the environment.

## Supporting information

S1 Table Elemental composition of humic or fulvic acids (%) and their C/N and C/H atomic ratios. (DOCX)

S2 Table Distribution (%) of carbon functions in CPMAS-^13^C-NMR spectra of humic samples obtained as reported in materials and methods. The hydrophobic index (HB) was obtained using the formula: HB index = δ(alkyl+aromatic C)/δ(O-alkyl+carboxylic C).
(DOCX)

S1 Fig AFM surface morphology of MoPrP in complex with HAGw and HALe. (TIF)

S2 Fig The region of RCSMs resonances in the ^1^H NMR spectrum of MoPrP(23–230) in the presence of increasing concentrations of zinc: in black, MoPrP(23–230) alone; in red MoPrP(23–230) with 100 μM ZnCl_2_; in green, MoPrP(23–230) with 300 μM ZnCl_2_; in violet, MoPrP(23–230) with 1 mM ZnCl_2_. (TIF)

S3 Fig Diffusion Ordered NMR spectra (DOSY NMR) experiments on MoPrP(23–230) alone (A) and in the presence of increasing Zn^2+^ concentrations: 100 μM (B), 300 μM (C) and 600 μM (D).
(TIF)
