# Supplementary material for: The mechanisms of humic substances self-assembly with biological molecules: The case study of the prion protein
Source: PLoS One. 2017 Nov 21;12(11):e0188308. doi: 10.1371/journal.pone.0188308 (PMC5697873; doi:10.1371/journal.pone.0188308)
Supplement: S1 Table — (DOCX) [file pone.0188308.s001.docx]

|  | **N (%)** | **C (%)** | **H (%)** | **C/N (%)** | **C/H (%)** |
| --- | --- | --- | --- | --- | --- |
| **HAGw** | 2.62 ± 0.16 | 29.5 ± 3.29 | 4.26 ± 0.01 | 11.3 | 6.9 |
| **HAS** | 4.57 ± 0.57 | 36.73 ± 0.22 | 4.5 ± 0.03 | 8.0 | 8.2 |
| **HALe** | 1.75 ± 0.05 | 50.47 ± 2.57 | 3.86 ± 0.05 | 28.9 | 13.1 |
| **FAGw** | 0.91 ± 0.09 | 13.33 ± 2.91 | 3.98 ± 0.05 | 14.7 | 3.4 |
| **FAS** | 1.3 ± 0.03 | 28.53 ± 0.14 | 3.81 ± 0.19 | 21.9 | 7.5 |
| **FABw** | 3.21 ± 0.29 | 32.05 ± 0.89 | 4.92 ± 0.08 | 10.0 | 6.5 |
