# Supplementary material for: The mechanisms of humic substances self-assembly with biological molecules: The case study of the prion protein
Source: PLoS One. 2017 Nov 21;12(11):e0188308. doi: 10.1371/journal.pone.0188308 (PMC5697873; doi:10.1371/journal.pone.0188308)
Supplement: S2 Table — The hydrophobic index (HB) was obtained using the formula: HB index = δ(alkyl+aromatic C)/δ(O-alkyl+carboxylic C). (DOCX) [file pone.0188308.s002.docx]

| **Chemical shift intervals** | **FABw** | **FAGw** | **FAS** | **HALe** | **HAGw** | **HAS** |
| --- | --- | --- | --- | --- | --- | --- |
| Carboxylic C (160-190 ppm) | 7.92 | 5.92 | 9.50 | 5.75 | 6.65 | 10.57 |
| Aromatic C (110-160 ppm) | 12.85 | 17.95 | 25.73 | 49.93 | 30.33 | 20.12 |
| O-Alkyl C (60-110 ppm) | 50.76 | 39.79 | 30.64 | 4.46 | 24.01 | 33.64 |
| Methoxyl C and C-N (45-60 ppm) | 10.89 | 12.84 | 11.38 | 4.31 | 11.51 | 12.53 |
| Alkyl C (0-45 ppm) | 17.59 | 23.50 | 22.75 | 35.54 | 27.50 | 23.13 |
| Hydrophobicity index (HB)^a^ | 0.52 | 0.91 | 1.21 | 8.37 | 1.89 | 0.98 |
| ^a^ [(0 - 45) + (110 - 160)]/[(60 - 110) + (160 - 190)] | | | | | | |
