# Supplementary figures and images for: The mechanisms of humic substances self-assembly with biological molecules: The case study of the prion protein
Source: PLoS One. 2017 Nov 21;12(11):e0188308. doi: 10.1371/journal.pone.0188308 (PMC5697873; doi:10.1371/journal.pone.0188308)

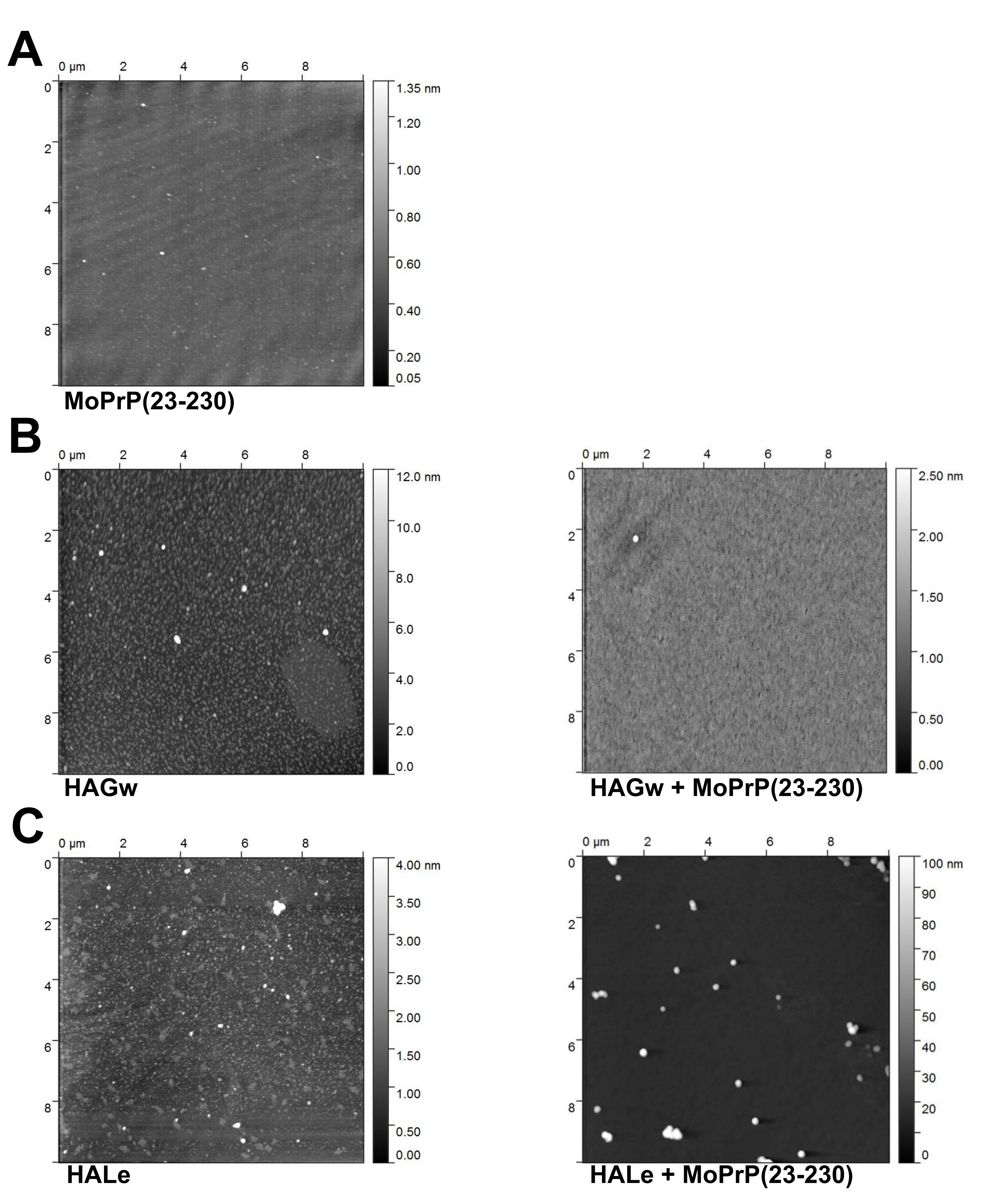

Supplement: S1 Fig — (TIF) [file pone.0188308.s003.tif]

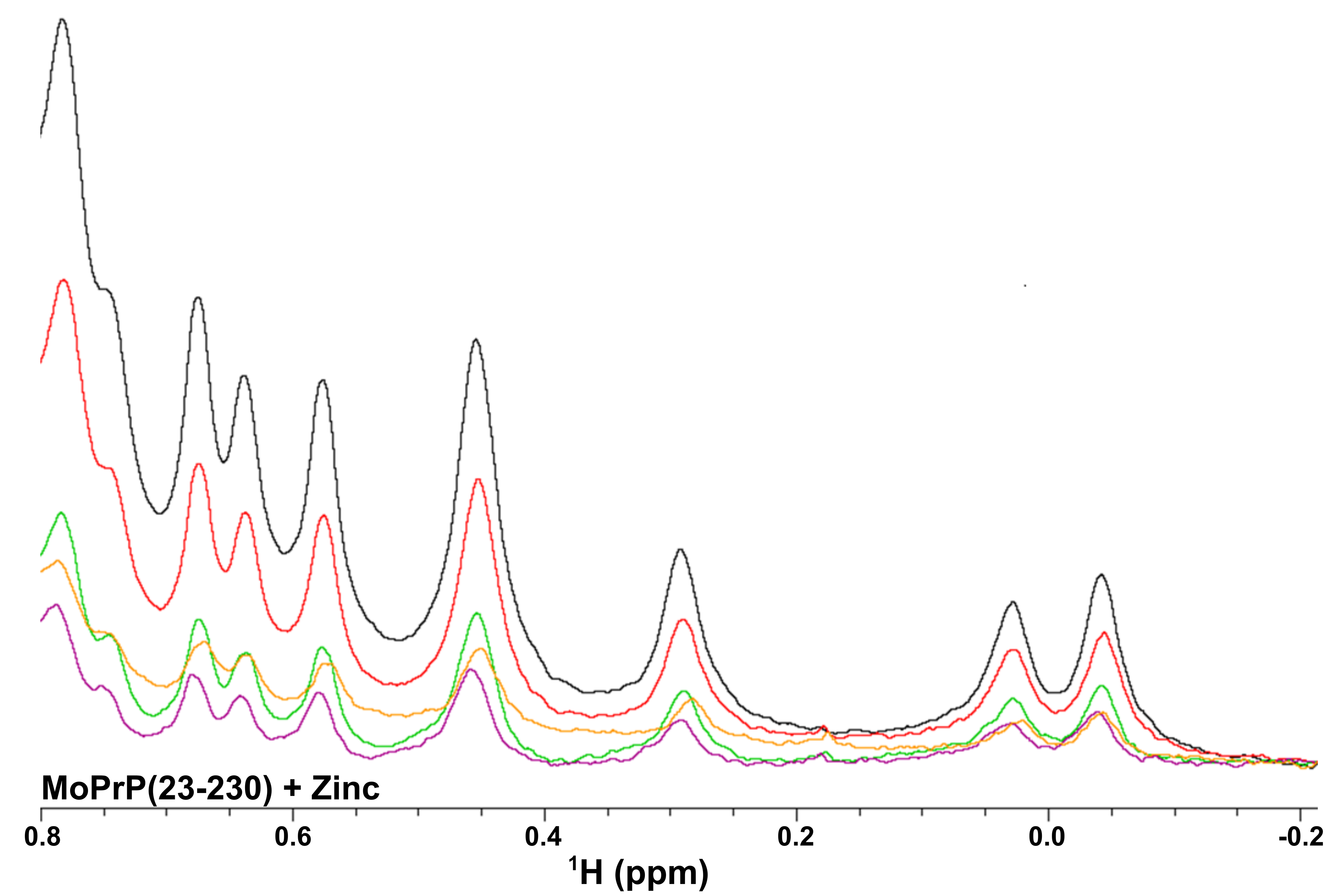

Supplement: S2 Fig — (TIF) [file pone.0188308.s004.tif]
